# Early peripheral blood lymphocyte subsets and cytokines in predicting the severity of influenza B virus pneumonia in children

**DOI:** 10.3389/fcimb.2023.1173362

**Published:** 2023-05-12

**Authors:** Lu Ma, Jingli Yan, Wenliang Song, Bo Wu, Zeyu Wang, Wei Xu

**Affiliations:** Department of Pediatrics, Shengjing Hospital of China Medical University, Shenyang, Liaoning Province, China

**Keywords:** Influenza B virus, pneumonia, children, cytokine, lymphocyte subsets

## Abstract

**Background:**

Children with influenza B virus infection have a higher susceptibility and higher severity of illness. The activation and disorder of immune function play an important role in the severity of influenza virus infection. This study aims to investigate whether early lymphocyte count and cytokines can provide predictive value for the progression in children with influenza B virus pneumonia.

**Methods:**

A retrospective cohort study was conducted to analyze the clinical data of children with influenza B virus pneumonia from December 1, 2021, to March 31, 2022, in the National Children’s Regional Medical Center (Shengjing Hospital of China Medical University). According to the severity of the disease, the children were divided into a mild group and a severe group, and the clinical characteristics, routine laboratory examination, lymphocyte subsets, and cytokines were compared.

**Results:**

A total of 93 children with influenza B virus pneumonia were enrolled, including 70 cases in the mild group and 23 cases in the severe group. Univariate analysis showed that drowsiness, dyspnea, white blood cell (WBC), lymphocytes, monocytes, procalcitonin, alanine aminotransferase (ALT), aspartate aminotransferase (AST), creatine kinase-MB (CK-MB), lactate dehydrogenase (LDH), fibrinogen (FIB), Immunoglobulin M (IgM), lung consolidation, total T cell count, CD4^+^ T cell count, CD8^+^ T cell count, NK cell count, NK cell % and B cell % had statistical differences between the mild and severe groups (*P*<0.05). In multivariate logistic regression analysis, reduced ALT (OR = 1.016), FIB (OR = 0.233), CD8^+^ T cell count (OR = 0.993) and NK cell count (OR = 0.987) were independently associated with the development of severe influenza B virus pneumonia.

**Conclusions:**

The levels of T lymphocytes and NK cells were related to the progression of influenza B virus pneumonia in children, and the reduction of CD8^+^ T cell count and NK cell count can be used as independent risk factors for predicting the severity of influenza B virus pneumonia.

## 1 Introduction

The influenza virus is one of the most common pathogens of respiratory infectious diseases. Globally, the annual incidence of influenza in children is estimated at 20% to 30%, and about 28,000 children under 18 years of age die of influenza-related lower respiratory tract infections, with most deaths occurring in children under 4 years of age (Park and Ryu, 2018; Troeger et al., 2019). Among all types of influenza viruses, influenza A and B are particularly common. We usually refer to the peak of influenza virus infection in the year as influenza season. In Northern hemisphere countries, influenza A infection peaks in January and February, and influenza B infection peaks in February and March (Bhat, 2020). Compared with the influenza A virus, the influenza B virus is rarer and usually occurs locally or seasonally rather than causing a pandemic. However, the influenza B virus is highly contagious and causes more severe disease and higher mortality than the influenza A virus (Gavigan and McCullers, 2019). Epidemiological and clinical studies on the influenza B virus mostly focus on adult patients, but children have a higher susceptibility to the influenza B virus and a greater risk of complications, hospitalization, and death (Paul Glezen et al., 2013; Koutsakos et al., 2016; Nayak et al., 2021).

The most common complication for hospitalized patients with influenza virus infection is pneumonia (Kalil and Thomas, 2019), the typical pathological changes are interstitial pneumonia, and severe patients may have lung parenchymal damage. Characteristic alveolar pathological changes of severe influenza virus pneumonia include capillary thrombosis, focal necrosis, congestion of alveolar walls, inflammatory infiltration, hyaline membrane formation, and pulmonary edema. Acute respiratory distress syndrome (ARDS) secondary to influenza greatly increases the difficulty of treatment, and even requires extracorporeal life membrane oxygenation (ECMO) treatment based on invasive mechanical ventilation, along with a higher mortality rate, heavier social burden, and increases serious pulmonary sequelae. In addition to direct damage to the airway and alveolar epithelium through the inherent pathogenicity of the influenza virus, the involvement of a strong immune response is also an important aspect of aggravating lung injury (Herold et al., 2015). The influenza virus first infects alveolar or airway epithelial cells. Viral RNA in the cytoplasm is recognized by RIG-I, TLR3, TLR7 and NLR to activate the innate immune response, and the TLR pathway activates downstream IRF3 and IRF7 to regulate IFN production. Viral RNA binds to the RIG-I receptor, triggering its interaction with MAVS to activate NF-κB, and MAVS and NLR activate the inflammasome to release IL-1β and IL-18 in response to viral RNA, resulting in the formation of cytokine storm (Gu et al., 2021; Keskinidou et al., 2022), which directly damages the pulmonary capillary endothelium and participates in the formation of pathological changes. The activation of adaptive immune response contributes to the clearance of the virus, while excessive immune response will induce excessive production of pro-inflammatory factors. The balance of pro-inflammatory and anti-inflammatory cytokines is particularly important for the disease’s incidence, severity, and mortality.

In the epidemic season of 2021-2022, there was an epidemic of influenza B among children in Northeast China, a high proportion of hospitalized influenza-like patients had pneumonia, and some patients had serious extrapulmonary complications. Therefore, we conducted a retrospective cohort study to investigate whether the early lymphocyte subsets counts and cytokines levels in the blood of children with influenza B virus pneumonia could provide a solid base for predicting the development of the disease.

## 2 Materials and methods

### 2.1 Population

**Figure 1:**
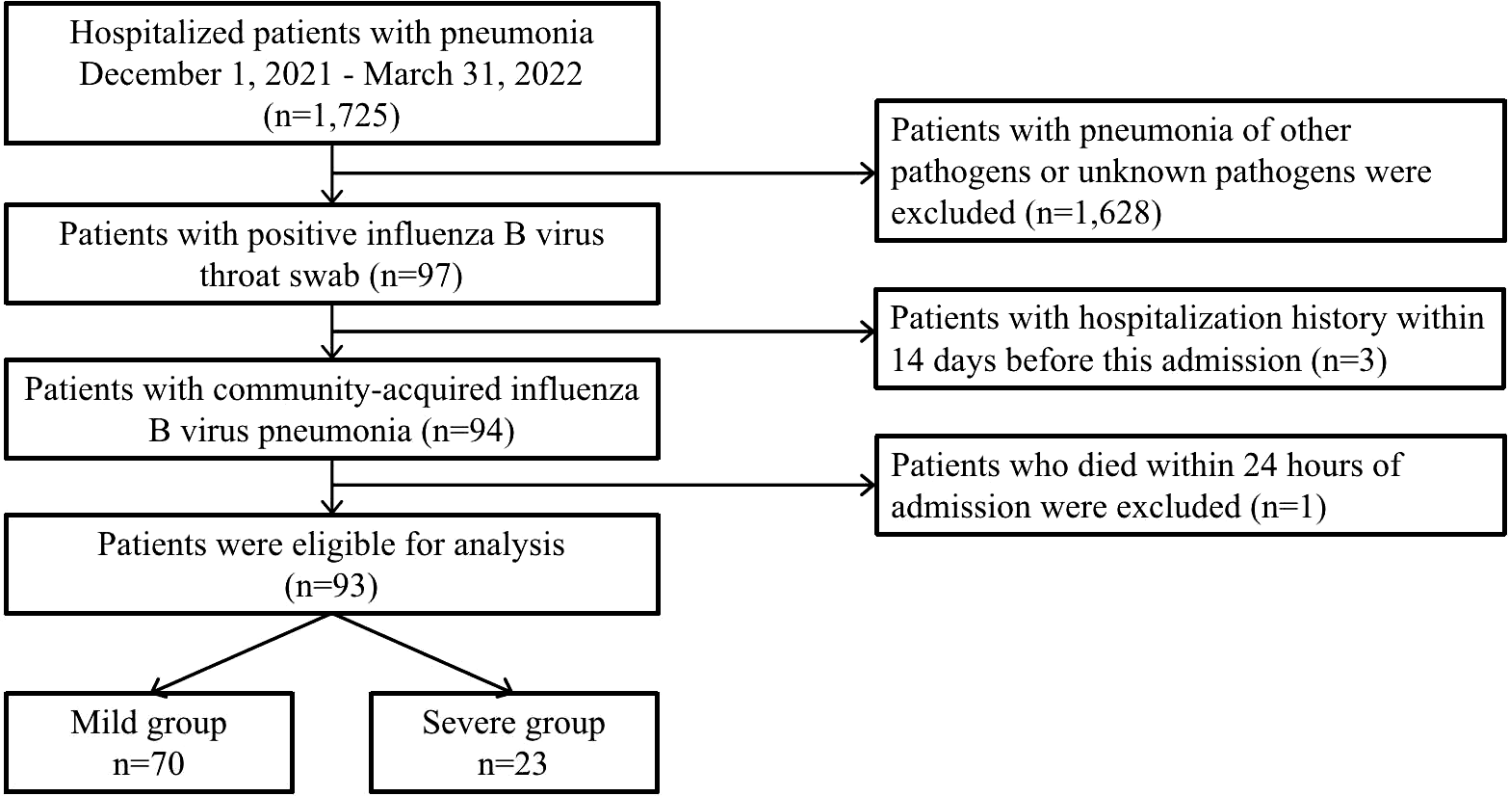
Flow chart of the study design and enrollment.

This study was a retrospective cohort study, patients’ information was collected through the Hospital Information System (HIS). This study was approved by the Ethics Committee of Shengjing Hospital of China Medical University (Approval number: 2022PS1030K). We enrolled patients (28 days to 14 years old) hospitalized with acute lower respiratory tract infection symptoms and diagnosed with influenza B virus pneumonia from December 1, 2021, to March 31, 2022, in the National Children’s Regional Medical Center (Shengjing Hospital of China Medical University). Pneumonia is defined as the presence of fever (temperature>37.5°), acute respiratory symptoms (cough, shortness of breath, dyspnea) or both, and new infiltration shadow or consolidation not attributable to other causes on chest imaging (McIntosh, 2002). Influenza virus infection is defined as the presence of influenza-like symptoms during influenza season and a positive throat swab for influenza B virus (Uyeki et al., 2019). Inclusion criteria: (1) Meet the diagnostic criteria for pneumonia. (2) Meet the etiological diagnostic criteria for clinical diagnosis of influenza B virus pneumonia. Exclusion criteria: (1) Patients with primary immunodeficiency and secondary immunodeficiency, such as long-term immunosuppressants and malignant tumors; (2) Patients with hospitalization history within 14 days before this admission; (3) The length of hospitalization is less than 24 hours. Evaluation criteria for severity of influenza B virus pneumonia: the patient who meets the above inclusion and exclusion criteria, combined with one or more of the following complications is defined as severe influenza B virus pneumonia, including acute respiratory failure, acute respiratory distress syndrome, secondary bacterial pneumonia, sepsis, myositis, rhabdomyolysis, acute myocarditis, acute pericarditis, acute encephalitis, acute disseminated encephalomyelitis, transverse myelitis, aseptic meningitis, and Guillain Barre syndrome (Chow et al., 2019; Kalil and Thomas, 2019), the remaining patients are defined as mild influenza B virus pneumonia. The study flow chart is shown in **Figure 1**.

### 2.2 Measurements

Nasopharyngeal swabs were obtained within 24 hours after admission, and influenza B virus RNA was detected in exfoliated cells by polymerase chain reaction (PCR) or nucleic acid hybridization. Venous blood samples were collected within 24 hours after admission, flow cytometry analysis was used to detect the absolute count and percentage of the total T cell (CD3^+^), CD4^+^ T cell (CD3^+^CD4^+^), CD8^+^ T cell (CD3^+^CD8^+^), NK cell (CD16^+^CD56^+^), the total B cell (CD19^+^) on the surface of lymphocytes, and the ratio of CD4^+^/CD8^+^ (Th/Ts). We used BD™ Cytometric Bead Array (CBA)Kit based on flow cytometry detected cytokines (IL-2, IL-4, IL-6, IL-10, IL-17, INF-γ, IL-12p70, IL-1β, TNF-α) in peripheral blood.

### 2.3 Statistical analysis

The SPSS software (V26.0, IBM, New York, USA) was adopted for statistical analysis. Normally distributed continuous variables were expressed as mean ± standard deviation (x ± s) and compared with a t-test. Non-normally distributed continuous variables were expressed as the median value (P25, P75), and the Mann–Whitney U rank sum test was used to compare the two groups. Categorical data were presented as n (%) and compared using a Chi-square test. Logistic regression analysis was performed to test the risk factors. *P* < 0.05 was considered statistically significant.

## 3 Results

### 3.1 Clinical characteristics and radiologic findings

93 children diagnosed with influenza B virus pneumonia were divided into a mild group (n = 70) and a severe group (n = 23). There were no statistical differences in gender and age between the mild and severe groups (*P*>0.05). There were differences in the types of underlying diseases between the two groups. The proportion of patients with congenital heart disease in the severe group was higher than that in the mild group (*P*<0.05, **Table 1**).

**Table 1:** Analysis of clinical characteristics and radiologic findings between the mild and severe group in influznza virus B pneumonia.

Variables	Mild (n=70)	Severe (n=23)	*P* value
Age [M(P_25_,P_75_)]	36.00 (24.75,72.00)	48.00 (24.00,72.00)	0.72
Gender [male(%)]	32 (45.7%)	9 (39.1%)	0.58
Febrile days [M(P_25_,P_75_)]	8.00 (5.00,11.00)	6.00 (4.00,10.00)	0.17
T_MAX_ (°C)[M(P_25_,P_75_)]	39.3 (39.0,39.8)	39.5 (38.7,39.5)	0.39
Length of stay in hospital [M(P_25_,P_75_)]	8.00 (7.00,10.00)	11.00 (9.00,16.00)	<0.05
Total course of disease [M(P_25_,P_75_)]	17.00 (14.75,24.00)	19.00 (12.00,24.00)	0.77
Underlying disease [n(%)]	4 (5.7%)	5 (21.7%)	0.06
Neurological diseases [n(%)]	3 (75%)	1 (25%)	1
Congenital heart disease [n(%)]	0 (0%)	3 (100%)	<0.05
Chronic lung disease [n(%)]	0 (0%)	1 (100%)	0.25
Endocrine system diseases [n(%)]	1 (100%)	0 (0%)	1
Fever [n(%)]	69 (98.6%)	21 (91.3%)	0.15*
Cough [n(%)]	68 (97.1%)	20 (87.0%)	0.18
Ruuny nose [n(%)]	9 (12.9%)	1 (4.3%)	0.45
Tachypnea [n(%)]	11 (15.7%)	3 (13.0%)	1.00
Drowsiness [n(%)]	4 (5.7%)	10 (43.5%)	<0.05
Diarrhoea [n(%)]	3 (4.3%)	4 (17.4%)	0.11
Dash [n(%)]	2 (2.9%)	0 (0.0%)	1.00
Dyspnea[n(%)]	2 (2.9%)	6 (26.1%)	<0.05
Crackles [n(%)]	37 (52.9%)	13 (56.5%)	0.76
Stridor [n(%)]	10 (14.3%)	5 (21.7%)	0.61
Laboratory examination			
WBC (x10^9^/L)[M(P_25_,P_75_)]	6.18 (4.44,7.61)	4.40 (2.14,6.04)	<0.05
Neutrophil (x10^9^/L)[M(P_25_,P_75_)]	3.20 (1.40,4.90)	1.80 (1.10,2.70)	0.09
Lymphocytes (x10^9^/L)[M(P_25_,P_75_)]	2.00 (1.40,3.20)	1.10 (0.80,2.30)	<0.05
Monocytes (x10^9^/L)[M(P_25_,P_75_)]	0.60 (0.40,0.90)	0.40 (0.10,0.60)	<0.05
CRP (mg/L)[M(P_25_,P_75_)]	5.69 (2.08,16.10)	5.29 (1.90,9.15)	0.48
PCT (ng/ml)[M(P_25_,P_75_)]	0.15 (0.08,0.34)	0.36 (0.15,1.05)	<0.05
ALT (U/L)[M(P_25_,P_75_)]	14.0 (10.8,21.0)	43 (17.5,216.5)	<0.05
AST (U/L)[M(P_25_,P_75_)]	31.5 (25.0,40.0)	146.0 (42.5,269.0)	<0.05
Creatinine (umol/L)[M(P25,P75)]	28.5 (23.2,32.3)	24.6 (22.1,30.5)	0.19
Urea (mmol/L)[M(P25,P75)]	2.9 (2.2,3.5)	2.9 (2.2,4.5)	0.38
CKMB (U/L)[M(P_25_,P_75_)]	21.0 (17.0,24.0)	25.0 (20.0,41.3)	<0.05
LDH (U/L)[M(P_25_,P_75_)]	293.5 (263.5,359.3)	744.5 (322.0,1424.0)	<0.05
Albumin (g/L)[M(P_25_,P_75_)]	38.5 (36.3,40.6)	34.7 (32.1,40.6)	<0.05
PT (s)[M(P_25_,P_75_)]	11.4 (10.8,12.5)	10.7 (10.3,11.4)	2.44
APTT (s) x ± s	35.0 (33.0,39.0)	36.0 (32.0,43.0)	0.56
FIB (g/L)[M(P_25_,P_75_)]	2.95 (2.68,3.58)	2.30 (1.43,3.05)	<0.05
D-Dimer(ug/L)[M(P_25_,P_75_)]	184.5 (116.8,237.3)	223.0 (139.3,604.3)	0.39
Immunoglobulin			
IgG (g/L)[M(P_25_,P_75_)]	7.94 (6.29,9.06)	7.39 (4.83,8.84)	0.22
IgA (g/L)[M(P_25_,P_75_)]	0.86 (0.50,1.36)	0.81 (0.50,1.35)	0.51
IgM (g/L)[M(P_25_,P_75_)]	1.08 (0.82,1.44)	0.76 (0.49,0.99)	<0.05
Oxygen or respiratory support	3 (4.3%)	14 (60.9%)	<0.05
Lung imaging characteristics			
unilateral	14 (20.0%)	3 (13.0%)	0.66
bilateral	56 (80.0%)	20 (87.0%)	0.66
patchy shadowing	57 (81.4%)	19 (82.6%)	1.00
stripe shadowing	12 (17.1%)	4 (17.4%)	1.00
nodules	8 (11.4%)	3 (13.0%)	1.00
consolidation	18 (25.7%)	14 (60.9%)	<0.05
multiple segments or lobes	6 (8.6%)	6 (26.1%)	0.07
hydrothorax	1 (1.4%)	4 (17.4%)	<0.05

∗Pearson Chi-squared test or Fisher exact test when appropriate.

T_MAX_, the maximum body temperature during the course of the disease; WBC, White blood cells; CRP, C-reactive protein; PCT, procalcitonin; ALT, alanine aminotransferase; AST, aspartate aminotransferase; CKMB, creatine kinase-MB; LDH, lactate dehydrogenase; PT, prothrombin time; APTT, activated partial thromboplastin time; FIB, fibrinogen; IgG, Immunoglobulin G; IgA, Immunoglobulin A; IgM, Immunoglobulin M.

Oxygen or respiratory support: nasal cannula oxygen, high-flow mask oxygen, non-invasive mechanical ventilation, invasive mechanical ventilation.

All the children had respiratory symptoms as the main clinical manifestations, including fever, cough, runny nose, and tachypnea. In addition to respiratory symptoms, a few patients combined with drowsiness, diarrhea, or rash. Among 93 children with influenza B virus pneumonia in our study, there were 5 cases of co-infection with pathogenically confirmed bacteria. 6 cases were complicated with acute encephalitis. 3 cases were complicated with hemophagocytic syndrome. 11 cases suffered from acute respiratory failure (8 cases of type I respiratory failure, and 3 cases of type II respiratory failure), including 5 cases with ARDS. Of the 93 patients, 18 were admitted to Pediatric intensive care unit (PICU), and 3 died. All 3 deaths were due to respiratory failure caused by infection exacerbation of the primary disease.

White blood cells (WBC), lymphocytes, monocytes, albumin, fibrinogen (FIB), and Immunoglobulin M (IgM) in the severe group were significantly lower than those in the mild group (*P*<0.05). The levels of procalcitonin (PCT), alanine aminotransferase (ALT), aspartate aminotransferase (AST), creatine kinase-MB (CK-MB), and lactate dehydrogenase (LDH) in the severe group were higher than those in the mild group (*P*<0.05). There were no significant differences in the levels of C-reactive protein (CRP), prothrombin time (PT), activated partial thromboplastin time (APTT), D-Dimer, creatinine, urea, Immunoglobulin G (IgG), and Immunoglobulin A (IgA) levels between the two groups (*P*>0.05). Lung imaging showed bilateral pneumonia was more common than unilateral pneumonia. The proportion of lung consolidation in the severe group was higher (*P*<0.05). The median length of stay in hospital in the severe group was longer than that in the mild group (*P*<0.05), and no statistical differences in the total course of disease, febrile days, and maximum temperature during the course of illness (*P*>0.05). As shown in **Table 1**.

### 3.2 Lymphocyte subsets

The absolute count of total T cell, CD4^+^ T cell, CD8^+^ T cell, and NK cell in the severe group was significantly lower than those in the mild group (*P*<0.05). There was no significant difference in total B cell count and CD4^+^/CD8^+^ ratio between the two groups (*P*>0.05). Our study showed that the percentage of total T cell, CD4^+^ T cell, and CD8^+^ T cell were no statiscally difference between the two groups (*P*>0.05), but the percentage of NK cell in the severe group was lower than that in the mild group, the percentage of total B cell was higher (*P*<0.05). As shown in **Table 2**.

**Table 2:** Comparisons of lymphocyte subsets between the mild and severe group in influznza virus B pneumonia.

Variables	Mild (n=70)	Severe (n=23)	*Z/t* value	*P* value
total T-cell Count (n/ul)[M(P_25_,P_75_)]	1614.0 (1168.0,2057.0)	800.0 (440.5,1521.5)	3.19	<0.05
total B-cell Count (n/ul)[M(P_25_,P_75_)]	535.0 (316.0,798.0)	409.0 (174.5,728.0)	1.61	0.11
CD4^+^ T-cell Count (n/ul)[M(P_25_,P_75_)]	878.0 (601.0,1156.0)	378.0 (251.5,724.5)	3.01	<0.05
CD8^+^ T-cell Count (n/ul)[M(P_25_,P_75_)]	562.0 (374.0,791.0)	301.0 (214.5,467.5)	3.91	<0.05
NK cell Count (n/ul)[M(P_25_,P_75_)]	206.0 (128.0,320.0)	88.0 (44.0,163.0)	4.22	<0.05
CD4^+^/CD8^+^ T-cell [M(P_25_,P_75_)]	1.62 (1.18,1.96)	1.39 (0.91,2.45)	0.85	0.39
total T-cell % x ± s	63.65 ± 11.02	60.68 ± 12.05	1.05	0.30
CD8^+^ T-cell % x ± s	23.55 ± 6.56	23.40 ± 8.01	0.88	0.93
CD4^+^ T-cell % x ± s	35.42 ± 8.90	33.88 ± 11.94	0.54	0.59
NK cell % [M(P_25_,P_75_)]	8.36 (6.33,13.10)	6.86 (4.94,8.57)	2.34	<0.05
total B-cell % x ± s	23.73 ± 10.41	30.26 ± 11.58	2.44	<0.05

NK cell, Natural killer cell.

### 3.3 Cytokines

The levels of cytokines IL-2, IL-4, IL-6, IL-10, IL-17, INF-γ, IL-12p70, IL-1β, and TNF-α were similar between the mild group and the severe group (*P*>0.05, **Table 3**).

**Table 3:** Comparisons of cytokines between the mild and severe group in influznza virus B pneumonia.

Variables	Mild (n=70)	Severe (n=23)	Z value	*P* value
IL-2 (pg/ml)[M(P_25_,P_75_)]	1.36 (1.07,2.01)	1.37 (1.08,1.96)	0.07	0.94
IL-4 (pg/ml)[M(P_25_,P_75_)]	1.98 (1.34,2.02)	1.89 (1.36,2.21)	1.18	0.86
IL-6 (pg/ml)[M(P_25_,P_75_)]	17.52 (7.14,40.10)	27.89 (13.89,91.66)	1.87	0.06
IL-10 (pg/ml)[M(P_25_,P_75_)]	3.79 (2.36,7.92)	5.82 (2.49,16.78)	1.24	0.22
IL-17 (pg/ml)[M(P_25_,P_75_)]	4.05 (2.47,7.29)	7.88 (3.81,10.95)	1.62	0.11
INF-γ (pg/ml)[M(P_25_,P_75_)]	7.95 (3.36,18.87)	9.52 (5.49,45.15)	0.91	0.36
IL-12p70 (pg/ml)[M(P_25_,P_75_)]	1.42 (1.02,1.76)	1.43 (1.04,2.06)	0.29	0.77
IL-1β (pg/ml)[M(P_25_,P_75_)]	1.35 (1.35,2.28)	1.35 (1.35,7.46)	0.44	0.66
TNF-α (pg/ml)[M(P_25_,P_75_)]	2.07 (1.04,2.12)	1.62 (1.09,2.82)	0.31	0.76

### 3.4 The multivariate logistic regression analysis for developing severe influenza B virus pneumonia

We excluded the factors by performing collinearity diagnostics on the predictors that were statistically significant in the univariate analysis, and finally included WBC, PCT, FIB, Albumin, ALT, CKMB, IgM, the percentage of NK cell and total B cell, CD8^+^ T cell count, CD4^+^ T cell count and NK cell count to conduct a multivariate logistic regression analysis, using the method of “back wald”. It revealed that ALT (OR = 1.016) was an independent risk factor for developing severe influenza B pneumonia, while CD8^+^ T cell count (OR = 0.993), FIB (OR = 0.233) and NK cell count (OR = 0.987) were protective factors (**Table 4**).

**Table 4:** The multivariate logistic regression analysis for developing severe influenza B virus pneumonia.

Variables	*OR*	95%*CI*	*P* value
FIB	0.233	0.065-0.835	<0.05
ALT	1.016	1.004-1.028	<0.05
CD8^+^ T-cell Count	0.993	0.989-0.998	<0.05
CD4^+^ T-cell Count	1.002	1.000-1.004	0.05
NK cell Count	0.987	0.974-1.002	<0.05

using method of “back wald”.

## 4 Discussion

Cellular and humoral immunity plays a key role in the body’s defense against viral infections, and activation and disruption of immune function have a significant impact on disease progression and prognosis. Our findings revealed that dysfunction in cellular immunity, especially changes in T lymphocytes and NK cells, were associated with the severity of the disease. As a key component of the immune system, CD4^+^ T cells significantly impact lung inflammation and lung injury caused by influenza virus infection by producing a large number of cytokines and coordinating with other immune and non-immune cells (Zens and Farber, 2015). CD4^+^ T cells stimulate B cell activation and differentiation. CD4^+^ T cells also assist in the differentiation of CD8^+^ T cells into cytotoxic effectors and memory cells, as well as the localization of memory CD8^+^ T cells in the infected respiratory tract. Furthermore, cytotoxicity, which has the potential to directly eliminate infected cells, is an increasingly proven function of CD4^+^ T cells (Swain et al., 2006; Sant et al., 2018). CD8^+^ cytotoxic T cells recognize virus-infected cells, induce apoptosis, and produce proinflammatory cytokines to inhibit viral replication, such as IFN-γ (Piet et al., 2011). The absolute counts of total T cell, CD4^+^ T cell and CD8^+^ T cell in the severe disease group were lower than those in the mild disease group. Furthermore, our results showed no statistical difference in the CD4^+^/CD8^+^ T cell ratio between the mild and severe groups, that is, CD4^+^ and CD8^+^ T cell decreased proportionally. Numerous studies on the influenza A virus have shown that a decrease in peripheral blood leukocytes, lymphocytes and lymphocyte subsets is an immune process of the body in the early stages of the disease (Chiappini et al., 2011; Cheng et al., 2019; Shi et al., 2019). Early studies have also shown that total lymphocyte count, CD3^+^, CD4^+^, and CD8^+^ T cell were significantly decreased in the acute phase in adults with influenza B virus pneumonia (Xu et al., 2013). Our study confirmed the same findings in children, in general agreement with the study of Ying Ding et al (Ding et al., 2013). In recent years, we have found that respiratory viral infections often trigger a decrease in lymphocytes in the peripheral blood. Previous studies have suggested that this may be related to virus-induced T cell destruction, B Liu et al. demonstrated that influenza A (H1N1) pdm09 infection can trigger thymus cell apoptosis and thymic atrophy (Liu et al., 2014). Whether the influenza B virus causes T lymphopenia in the same way, is not known, but this could be an alternative explanation for the predominance of T lymphocytopenia. However, it is now generally believed that this is due to the convergence of peripheral memory T cells to the lung after viral infection, causing a decrease in T lymphocytes in the peripheral blood.

During the 2009 pandemic, it was shown that specific CD8^+^ T cell levels were inversely correlated with disease severity caused by infection with the H1N1pdm09 virus (Sridhar et al., 2013). Taoran Geng et al. divided adult influenza patients into mild, severe, severe survival, and severe death groups, and found that the level of CD8^+^ T cell in severe patients was lower than those in mild patients (Geng et al., 2020), this is consistent with the results of our study. It suggests that CD8^+^ T cell play a key role in the body’s defense against influenza virus infection, and the results of our study revealed that CD8^+^ cell acted as an independent protective factor. Similarly, as a respiratory virus, the relationship between the severity of COVID-19 and the immune response is a focus of attention. A large number of studies have shown that CD4^+^, CD8^+^ T cell and NK cell were significantly reduced in COVID-19 patients, and were associated with COVID-19 severity and prognosis, and both CD8^+^ and CD4^+^ T cell served as diagnostic markers of COVID-19 and predictors of disease severity (Huang et al., 2020; Jiang et al., 2020).

One of the important functions of humoral immunity in influenza virus infection is antibody-mediated virus neutralization. Although previous studies have found that B lymphocytes decreased in adult patients after influenza B virus infection (Xu et al., 2013), the level of B lymphocytes was similar between the mild and severe groups in our study. In the total lymphocyte, the percentage of B lymphocytes in the severe group was higher than that in the mild group, this is a consequence of the greater reduction in T lymphocytes and NK cells in the severe group in our study. However, there is no report that it is related to the severity of the disease, and the comparison of B lymphocytes between children with influenza and healthy children needs to be further studied. Abira Paramsothy et al. reported that patients with severe influenza A H1N1 infection have high levels of functional humoral immune response and low levels of antibody affinity and proposed that antibody levels increase with the severity of the disease and speculated that high viral load can enhance the humoral immune response (Paramsothy et al., 2021). In contrast, we compared serum immunoglobulins and found that early IgM levels in the severe group were significantly lower than in the mild group, and we hypothesized that reduced early serum IgM led to a weakening of the neutralizing effect of the virus and aggravated the severity of the disease.

Natural killer (NK) cells are the early line of defense against influenza virus infection. Before the adaptive immune response initiation, NK cells not only produce antiviral-related cytokines but also directly participate in the rapid clearance of virus-infected cells and interact with dendritic cells to directly regulate the adaptive immune response (Jost et al., 2011). In a mouse model of influenza A virus infection, increased aggregation of NK cell in the lung can be observed, but the role of circulating and tissue NK cell in antiviral immunity is not yet fully explained (Björkström et al., 2022). During acute seasonal or pandemic H1N1 infections, a decrease in the absolute number of NK cell can be observed, and reduced or absent NK cell activity leads to delayed viral clearance and increased morbidity and mortality (Denney et al., 2010). It was shown that although the affinity of the influenza B virus for NKp46 was lower compared to the influenza A virus, it did not affect its clearance by NK cell (Duev-Cohen et al., 2020). Our results showed that in the early stage of influenza B virus pneumonia, the level of NK cells was significantly lower in the severe group. In addition, the percentage of NK cell was lower in the severe group, which makes our conclusions more convincing. The clinical symptoms were more prominent in the severe group, causing more organ dysfunction in addition to pneumonia, including 3 cases of the combined hemophagocytic syndrome, which is more evidence that NK cell plays an important role in the progression of influenza B virus infection.

Cytokines are involved in regulating the balance between humoral and cellular immune responses and in regulating the maturation, growth, and response of specific cell populations, IL-1β, IL-8, and IFN-γ are pro-inflammatory factors, which are involved in the early response and amplification of the inflammatory response, while anti-inflammatory cytokines such as IL-4, IL-10 and IL-13 limit the inflammatory response. The influenza virus is a pathogen with high morbidity and mortality, and excessive levels of pro-inflammatory cytokines are thought to be associated with extensive tissue damage and the development of influenza-related complications (Liu et al., 2016). Analysis of infected children during the 2009 influenza A (H1N1) virus pandemic revealed that levels of IFN-γ, IL-6, IL-8, IL-10, and other cytokines were higher in pneumonia patients than in non-pneumonia patients (Takano et al., 2011). Yu Xie et al (Xie et al., 2021) examined sputum specimens from patients with severe and non-severe influenza A virus pneumonia, found elevated levels of IL-6, IFN- γ, and IL-2 in patients with severe influenza A pneumonia and significantly higher levels of IL-2 and IL-6 in non-surviving patients than in survivors. This suggests that influenza virus infection triggers elevated cytokine levels and correlates with clinical disease severity and prognosis.

We studied IL-2, IL-4, IL-6, IL-10, IL-17, IFN-γ, and TNF-α and found that early cytokine levels were not significantly related to the severity of influenza B virus pneumonia, which is inconsistent with previous studies on cytokines in influenza A. Masatoki Sato et al. examined serum concentrations of IFN-c and IL-4 representing Th1 and Th2 cytokines in children with influenza and found that serum levels of IFN-c and IL-4 were higher in patients with influenza A than in those with influenza B. The high ratio of IL-4 to IFN-c and the predominance of Th2 cytokine production after influenza A suggest differences in the immune response system between influenza A and B infections (Sato et al., 2009). This may be one reason why our results were inconsistent with them. In addition, this may be related to the stage of our testing, as cytokine testing in children is usually performed within 24 hours of admission, while some patients were still in the progressive stage of the disease and not at the most severe point of the disease. Although the levels of cytokines in the early stage of influenza B virus pneumonia in our study were no statistical differences, the relationship between the levels of cytokines and the severity of influenza B virus pneumonia in children needs dynamic monitoring and to be further determined, which needs to be proved by dynamic clinical studies with large samples.

Our study showed that WBC in severe group was lower than that in mild group, and the overall decrease in leukocytes should be mainly contributed by lymphocytes. ALT, AST, CKMB, and LDH were significantly higher in severe group, which can indicate the existence of more extensive tissue damage in severe patients. It is related to the immune response and the intensity of inflammatory damage after infection and has certain implications on clinical recognition of the severity of influenza B virus pneumonia. CRP, a non-specific marker of inflammation, was similar between the two groups, this is consistent with previous study (Ahn et al., 2011). It has been reported that patients with bacterial co-infection had significantly higher serum CRP levels than patients with HIN1 infection alone (Ahn et al., 2011). In our study, although 5 children in the severe group had a bacterial infection, we tested CRP within 24 hours of admission, and it usually peaks at 24-48 hours. Procalcitonin is often used as a marker to distinguish non-infectious diseases from sepsis, but it is also associated with liver injury, central nervous system injury, and lung injury in addition to bacterial infection. In our study, procalcitonin in severe group was higher, which was not only related to secondary bacterial pneumonia in some cases but also suggested that the severe group was complicated with other tissue damage based on influenza B virus pneumonia. Influenza virus directly damages the endothelium of pulmonary capillaries and can cause coagulation dysfunction, DIC, and pulmonary embolism (Yang and Tang, 2016). Unlike the results of ferret model (Goeijenbier et al., 2014), the coagulation abnormalities in our study were not significant and the FIB was lower in severe group, which was considered that the increase of FIB means that the body’s stress state and vascular repair ability was stronger during severe infection. The pulmonary imaging of children with influenza B virus pneumonia was mostly bilateral pneumonia, and consolidation was more common in severe cases, and some of them complicated with hydrothorax, which is a differential point between influenza B virus pneumonia and COVID-19 pneumonia during the epidemic (Liu et al., 2020; Kuang et al., 2021).

Host factors are relevant to the severity of influenza illness, including underlying diseases such as heart disease, lung disease, or neurological disorders (Doyle and Campbell, 2019). A study of influenza B virus pneumonia in adults showed that chronic respiratory disease was independent risk factor for influenza B virus pneumonia in adult patients (Dai et al., 2020). Children with congenital heart disease were more likely to develop severe influenza B virus pneumonia in our study. Unfortunately, there were only 9 cases of children with underlying diseases, which failed to conduct multivariate logistic analysis. Although the number of deaths in our study was too small to analyze the risk factors for death, all 3 death cases suffered from underlying diseases. One of them combined with neuromuscular disease, it is consistent with the results of the study that chronic neurological diseases were independent risk factors for mortality of influenza A (H1N1) pdm09 in children (Chow et al., 2019). The COVID-19 pandemic has also shown that the risk of infection and mortality increases with the increase of the underlying diseases (Rahman et al., 2021). Previous studies have shown that influenza A virus infection combined with bacterial infection is a risk factor for death in children. In our cohort study, there were 5 cases of combined bacterial infections and no death cases. Whether influenza B virus pneumonia combined with bacterial infection is different from that of influenza A and does not increase the risk of death remains to be verified by large sample studies.

There are several limitations in our study. First, this is a small sample, single-center retrospective study, which may exist selection biases. Second, we lack comparisons of lymphocyte subsets and cytokines between children with influenza B virus pneumonia and healthy children. Finally, we lack data on dynamics throughout the course of the disease. The research on the changes in lymphocyte subsets and cytokines in children after influenza B virus infection remains to be in-depth.

## 5 Conclusion

Influenza B virus infection in children should not be underestimated, in whom pneumonia is common. Severe cases need intensive care, mechanical ventilation, and other active treatment measures. It is important to monitor the immune status of children with influenza B virus pneumonia in the early stage. Early declines in CD4^+^, CD8^+^ T cells, and NK cells were associated with the direction of disease progression, the depression of CD8^+^ T cell and NK cell were independent risk factors for the development of severe influenza B virus pneumonia.

## Data availability statement

The raw data supporting the conclusions of this article will be made available by the authors, without undue reservation.

## Ethics statement

The studies involving human participants were reviewed and approved by the Ethics Committee of Shengjing Hospital of China Medical University. Written informed consent from the participants’ legal guardian/next of kin was not required to participate in this study in accordance with the national legislation and the institutional requirements.

## Author contributions

LM drafted the manuscript. JY and ZW collected the data of patients. BW and WS analyzed and interpreted the data. WX was responsible for our study design and manuscript review. All authors contributed to the article and approved the submitted version.
